# Supplementary material for: Hot spot prediction in protein-protein interactions by an ensemble system
Source: BMC Syst Biol. 2018 Dec 31;12(Suppl 9):132. doi: 10.1186/s12918-018-0665-8 (PMC6311905; doi:10.1186/s12918-018-0665-8)
Supplement: Supplementary file 2 — All relevant MATLAB code. A simple Matlab implement of our predictor. (ZIP 19,742 kb) [file 12918_2018_665_MOESM2_ESM.zip › Additional file 2/Read ASA/ASA Property/SKEMPI/1KTZB.txt]

 NetSurfP - Protein Surface Accessibility and Secondary Structure Predictions

  

|  |  |  |
| --- | --- | --- |
|  |  | NetSurfP - Protein Surface Accessibility and Secondary Structure Predictions  Technical University of Denmark |

  


---

```
# For publication of results, please cite:
# A generic method for assignment of reliability scores applied to solvent accessibility predictions.
# Bent Petersen, Thomas Nordahl Petersen, Pernille Andersen, Morten Nielsen and Claus Lundegaard
# BMC Structural Biology 2009, 9:51 doi:10.1186/1472-6807-9-51
#
# Column 1: Class assignment - B for buried or E for Exposed - Threshold: 25% exposure, but not based on RSA
# Column 2: Amino acid
# Column 3: Sequence name
# Column 4: Amino acid number
# Column 5: Relative Surface Accessibility - RSA
# Column 6: Absolute Surface Accessibility
# Column 7: Z-fit score for RSA prediction
# Column 8: Probability for Alpha-Helix
# Column 9: Probability for Beta-strand
# Column 10: Probability for Coil
E P  1KTZB                  1    0.725 102.849  -0.572   0.003   0.003   0.994
E Q  1KTZB                  2    0.534  95.372   0.246   0.019   0.141   0.840
E L  1KTZB                  3    0.313  57.384   0.267   0.021   0.279   0.699
B C  1KTZB                  4    0.091  12.706  -0.628   0.021   0.279   0.699
E K  1KTZB                  5    0.548 112.785   0.636   0.066   0.296   0.638
E F  1KTZB                  6    0.353  70.767  -0.370   0.064   0.216   0.721
B C  1KTZB                  7    0.134  18.856   0.449   0.019   0.141   0.840
E D  1KTZB                  8    0.492  70.912   0.524   0.018   0.088   0.893
E V  1KTZB                  9    0.390  59.912  -0.999   0.018   0.047   0.935
E R  1KTZB                 10    0.618 141.591   0.172   0.018   0.047   0.935
E F  1KTZB                 11    0.399  79.979  -0.346   0.018   0.047   0.935
E S  1KTZB                 12    0.460  53.900  -0.905   0.018   0.047   0.935
E T  1KTZB                 13    0.450  62.470  -0.288   0.018   0.088   0.893
B C  1KTZB                 14    0.091  12.790  -1.399   0.018   0.088   0.893
E D  1KTZB                 15    0.669  96.374  -0.284   0.018   0.088   0.893
E N  1KTZB                 16    0.582  85.146  -1.447   0.018   0.047   0.935
E Q  1KTZB                 17    0.569 101.552   0.203   0.018   0.047   0.935
E K  1KTZB                 18    0.576 118.442  -0.653   0.019   0.141   0.840
E S  1KTZB                 19    0.314  36.801   1.005   0.022   0.552   0.426
B C  1KTZB                 20    0.074  10.446  -1.158   0.023   0.655   0.322
B M  1KTZB                 21    0.270  53.947  -0.646   0.022   0.552   0.426
B S  1KTZB                 22    0.266  31.128  -1.733   0.022   0.359   0.619
E N  1KTZB                 23    0.400  58.575  -1.165   0.021   0.279   0.699
B C  1KTZB                 24    0.065   9.070  -0.142   0.020   0.205   0.775
E S  1KTZB                 25    0.409  47.970  -0.418   0.021   0.451   0.528
B I  1KTZB                 26    0.122  22.478  -0.204   0.023   0.655   0.322
E T  1KTZB                 27    0.325  45.091  -0.217   0.021   0.756   0.223
B S  1KTZB                 28    0.268  31.433  -0.638   0.021   0.756   0.223
B I  1KTZB                 29    0.288  53.206   0.106   0.021   0.756   0.223
B C  1KTZB                 30    0.059   8.312   0.079   0.023   0.655   0.322
E E  1KTZB                 31    0.482  84.293  -0.304   0.021   0.279   0.699
E K  1KTZB                 32    0.404  83.041   0.459   0.005   0.045   0.951
E P  1KTZB                 33    0.502  71.262   0.116   0.018   0.019   0.964
E Q  1KTZB                 34    0.576 102.802  -0.173   0.053   0.043   0.903
E E  1KTZB                 35    0.343  59.922   0.868   0.066   0.296   0.638
B V  1KTZB                 36    0.046   7.147   1.233   0.021   0.756   0.223
B C  1KTZB                 37    0.030   4.142   0.784   0.011   0.918   0.071
B V  1KTZB                 38    0.044   6.686   0.765   0.006   0.962   0.032
B A  1KTZB                 39    0.033   3.670   0.085   0.006   0.962   0.032
B V  1KTZB                 40    0.034   5.164   0.444   0.006   0.962   0.032
B W  1KTZB                 41    0.057  13.612   0.600   0.006   0.962   0.032
B R  1KTZB                 42    0.231  52.945   0.821   0.018   0.846   0.136
E K  1KTZB                 43    0.409  84.049   0.858   0.023   0.655   0.322
E N  1KTZB                 44    0.622  91.032  -0.027   0.004   0.197   0.799
E D  1KTZB                 45    0.733 105.683  -1.325   0.005   0.015   0.979
E E  1KTZB                 46    0.694 121.242  -0.436   0.005   0.045   0.951
E N  1KTZB                 47    0.523  76.626  -0.307   0.005   0.262   0.733
B I  1KTZB                 48    0.151  27.991   0.437   0.004   0.616   0.381
E T  1KTZB                 49    0.300  41.652   0.951   0.001   0.900   0.099
B L  1KTZB                 50    0.107  19.592   0.147   0.011   0.918   0.071
B E  1KTZB                 51    0.246  43.064   0.004   0.011   0.918   0.071
B T  1KTZB                 52    0.114  15.853  -1.009   0.018   0.846   0.136
B V  1KTZB                 53    0.122  18.813   0.568   0.018   0.846   0.136
B C  1KTZB                 54    0.029   4.044  -0.160   0.023   0.655   0.322
B H  1KTZB                 55    0.212  38.490  -0.330   0.021   0.279   0.699
B D  1KTZB                 56    0.241  34.800  -0.036   0.005   0.045   0.951
B P  1KTZB                 57    0.225  31.913  -0.561   0.018   0.019   0.964
E K  1KTZB                 58    0.588 120.910  -0.052   0.018   0.047   0.935
B L  1KTZB                 59    0.242  44.237   0.868   0.053   0.043   0.903
E P  1KTZB                 60    0.257  36.525   0.287   0.052   0.084   0.864
B Y  1KTZB                 61    0.125  26.712  -0.457   0.118   0.150   0.732
B H  1KTZB                 62    0.279  50.786  -0.250   0.125   0.227   0.648
B D  1KTZB                 63    0.253  36.400  -2.035   0.135   0.317   0.548
B F  1KTZB                 64    0.115  23.121   0.668   0.069   0.386   0.545
B I  1KTZB                 65    0.145  26.788  -0.226   0.074   0.484   0.442
B L  1KTZB                 66    0.085  15.655   0.807   0.021   0.279   0.699
E E  1KTZB                 67    0.519  90.617  -0.132   0.019   0.141   0.840
E D  1KTZB                 68    0.449  64.643  -0.675   0.018   0.047   0.935
E A  1KTZB                 69    0.370  40.763  -1.287   0.018   0.019   0.964
E A  1KTZB                 70    0.623  68.644  -1.012   0.018   0.019   0.964
E S  1KTZB                 71    0.383  44.911  -0.474   0.018   0.047   0.935
E P  1KTZB                 72    0.516  73.164  -0.087   0.019   0.141   0.840
E K  1KTZB                 73    0.462  94.992   1.550   0.004   0.514   0.481
B C  1KTZB                 74    0.058   8.185  -0.145   0.002   0.816   0.182
E I  1KTZB                 75    0.288  53.298   0.342   0.001   0.900   0.099
B M  1KTZB                 76    0.148  29.675  -0.438   0.001   0.900   0.099
E K  1KTZB                 77    0.394  81.149   0.660   0.003   0.718   0.279
E E  1KTZB                 78    0.615 107.458   0.175   0.004   0.616   0.381
E K  1KTZB                 79    0.446  91.680   0.908   0.004   0.420   0.576
E K  1KTZB                 80    0.588 120.972  -0.972   0.005   0.262   0.733
E K  1KTZB                 81    0.476  98.016  -0.099   0.004   0.085   0.910
E P  1KTZB                 82    0.757 107.447  -1.004   0.018   0.019   0.964
E G  1KTZB                 83    0.674  53.060  -1.350   0.005   0.015   0.979
E E  1KTZB                 84    0.288  50.296  -0.542   0.004   0.085   0.910
E T  1KTZB                 85    0.250  34.730   0.905   0.004   0.514   0.481
B F  1KTZB                 86    0.064  12.785   0.695   0.001   0.900   0.099
B F  1KTZB                 87    0.064  12.885   0.495   0.001   0.959   0.040
B M  1KTZB                 88    0.059  11.786   0.472   0.001   0.959   0.040
B C  1KTZB                 89    0.019   2.654   0.766   0.001   0.900   0.099
B S  1KTZB                 90    0.119  13.912  -1.017   0.002   0.816   0.182
B C  1KTZB                 91    0.047   6.641   0.120   0.004   0.514   0.481
E S  1KTZB                 92    0.411  48.157   0.644   0.020   0.205   0.775
E S  1KTZB                 93    0.564  66.042  -1.041   0.052   0.084   0.864
E D  1KTZB                 94    0.598  86.172   0.019   0.052   0.084   0.864
E E  1KTZB                 95    0.531  92.713   0.373   0.056   0.142   0.802
B C  1KTZB                 96    0.080  11.288  -0.107   0.064   0.216   0.721
E N  1KTZB                 97    0.403  59.028  -0.091   0.020   0.205   0.775
B D  1KTZB                 98    0.309  44.541  -1.112   0.022   0.359   0.619
E N  1KTZB                 99    0.372  54.505   0.808   0.018   0.846   0.136
B I  1KTZB                100    0.096  17.834   0.680   0.011   0.918   0.071
B I  1KTZB                101    0.184  34.114  -0.055   0.018   0.846   0.136
B F  1KTZB                102    0.122  24.526  -0.010   0.021   0.756   0.223
E S  1KTZB                103    0.319  37.399  -0.516   0.021   0.451   0.528
E E  1KTZB                104    0.463  80.921  -0.753   0.021   0.279   0.699
E E  1KTZB                105    0.683 119.268  -0.359   0.019   0.141   0.840
E Y  1KTZB                106    0.618 132.152  -1.161   0.003   0.003   0.994

Explain the output.  Go back.
```

---
